# Nanobioelectrocatalysis Using Human Liver Microsomes and Cytochrome P450 Bactosomes: Pyrenyl-Nanocarbon Electrodes

**DOI:** 10.1021/acsabm.3c01170

**Published:** 2024-03-03

**Authors:** Gayan Premaratne, Jinesh Niroula, James T. Moulton, Sadagopan Krishnan

**Affiliations:** Department of Chemistry, Oklahoma State University, Stillwater, Oklahoma 74078, United States

**Keywords:** pyrenylamine, pi–pi stacking, amine-nanotubes, liver microsomes, P450, electrocatalysis, bioelectrodes

## Abstract

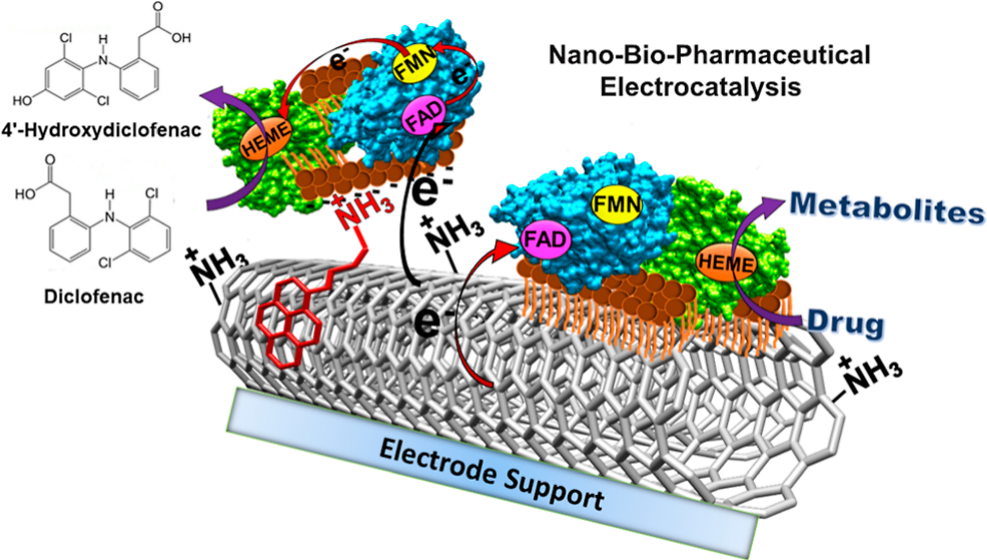

Human liver microsomes containing various drug-metabolizing cytochrome P450 (P450) enzymes, along with their NADPH-reductase bound to phospholipid membranes, were absorbed onto 1-pyrene butylamine pi–pi stacked with amine-functionalized multiwalled carbon nanotube-modified graphite electrodes. The interfaced microsomal biofilm demonstrated direct electrochemical communication with the underlying electrode surface and enhanced oxygen reduction electrocatalytic activity typical of heme enzymes such as P450s over the unmodified electrodes and nonenzymatic currents. Similar enhancements in currents were observed when the bioelectrodes were constructed with recombinant P450 2C9 (single isoform) expressed bactosomes. The designed liver microsomal and 2C9 bactosomal bioelectrodes successfully facilitated the electrocatalytic conversion of diclofenac, a drug candidate, into 4′-hydroxydiclofenac. The enzymatic electrocatalytic metabolite yield was several-fold greater on the modified electrodes than on the unmodified bulk graphite electrodes adsorbed with a microsomal or bactosomal film. The nonenzymatic metabolite production was less than the enzymatically catalyzed metabolite yield in the designed microsomal and bactosomal biofilm electrodes. To test the throughput potential of the designed biofilms, eight-electrode array configurations were tested with the microsomal and bactosomal biofilms toward electrochemical 4′-hydroxydiclofenac metabolite production from diclofenac. The stability of the designed microsomal bioelectrode was assessed using nonfaradaic impedance spectroscopy over 40 h, which indicated good stability.

## 1 Introduction

The process of drug development is a complex and time-consuming endeavor, which requires a thorough understanding of the metabolic pathways of drug candidates to ensure their efficacy and safety.^1,2^ In vitro drug metabolism using liver microsomal enzymes has been extensively studied and is essential in the development of drugs.^3−5^ Among the enzymes involved in liver microsomal biocatalysis, the cytochrome P450 (P450) enzyme system plays an important role in metabolizing drugs and xenobiotic compounds.^6−8^ The P450 enzymes are a family of heme-containing monooxygenases. These enzymes are membrane-bound and typically found in the endoplasmic reticulum of liver cells, where they are responsible for the oxidative metabolism of a wide range of drugs and other xenobiotics. The P450 enzyme system has multiple isoforms, each with a different substrate specificity and rate of metabolism.^9^ Some P450 isoforms also display genetic polymorphisms that can impact their metabolic and catalytic activities and contribute to individual differences in drug response and toxicity.^10−12^

While biochemical in vitro assays and methods are valuable, an alternate complementing analytical technique is to drive the purified P450 and microsomal biocatalysis electrochemically under an applied reductive potential with simplicity, creating a bioelectrocatalytic system.^13,14^ In this system, the P450 enzyme is attached to an electrode surface either in the purified form or bound to the microsomal membrane or other support materials.^15−20^ When a substrate is added to the system, the P450 enzyme catalyzes the oxidation of the substrate, generating an electrochemical signal that can be measured. In the same system, in the place of substrates, specific P450 inhibitors can be used to profile the activity of a specific P450 isoform toward a drug conversion into metabolites.^21^

The electrochemical driving of immobilized microsomal P450 electrocatalysis is a cost-effective, efficient probing of the enzymes and their reductase-mediated electron transfer pathway for the direct measurement of P450 activity and drug metabolism and inhibition assays.^19,22,23^ Moreover, the recent advancements allowing the successful direct use of human liver microsomes and bacterial-expressed-specific P450-isoform called bactosomes for bioelectrocatalysis have revolutionized the field significantly.^7,18,19^ By immobilizing the P450 enzyme onto an electrode surface, we can also create a highly sensitive and selective biosensor for the detection of drugs, environmental pollutants, and other xenobiotics.^21,24−26^

In recent years, nanomaterials-based liver microsomal cytochrome P450 electrocatalysis has emerged as a promising tool for enhanced production of metabolites with improved electrocatalytic activity on electrodes compared to conventional bulk electrode materials such as carbon and gold.^26,27^ Notable contributions from our laboratory include the integration of nanomaterials such as carbon nanotubes and magnetic nanoparticles into the liver microsomal cytochrome P450 electrocatalysis that significantly improved the analytical sensitivity, biofilm stability, and enhanced metabolite production compared to bulk electrodes without the nanomaterials use (e.g., graphite or gold disk electrodes).^7^ Moreover, these nanobioelectrodes can be miniaturized and automated, allowing for high-throughput screening of drug candidates, speeding up the drug development process, and reducing cost. Furthermore, electrochemical techniques offer several advantages over other types of biosensors such as optical biosensors.

Electrochemical biosensors are often more sensitive, selective, and stable than other types of biosensors, and they are also easier to integrate into microfluidic or lab-on-a-chip devices.^28−30^ Among various nanomaterials-modified bioelectrodes, the noncovalent functionalization of carbon nanostructures has gained considerable attention in biological electrocatalysis, energy devices, and sensors.^31−39^ The simplicity of making the surface modification, scalability, and surface stability of pyrenyl-nanocarbon-coated graphitic surface under rigorous conditions with retained enzyme activity (e.g., under an electrode rotation at high speeds such as 2000 rpm for 4 days)^32^ makes them very attractive for biological electrocatalysts, operating in aqueous electrolytes. Continuing our microsomal bioelectrocatalysis contributions, in the present work, we have designed a pi–pi-stacked amine-functionalized pyrenylbutylamine with the amine-functionalized carbon-nanostructures modified electrodes for stable electrostatic immobilization of human liver microsomes. The combined amine functionalization strategy (one from the nanostructures and the other from the pyrenyl pi–pi stackers) to facilitate electrostatic interactions with negatively charged lipid membranes of microsomes and additional strong hydrophobic interactions with the nonpolar tails of the lipid membranes is explored for the first time in this report.

## 2 Experimental Section

### 2.1 Materials and Methods

Human liver microsomes (HLM, mixed gender, pool of 50, suspension medium was 250 mM sucrose; XenoTech LLC, Lenexa, KS, USA) contained the following composition: Total protein was 20 mg mL^–1^, the total amount of P450 enzymes was 470 pmol mg^–1^ protein, NADPH-cytochrome c reductase activity was 181 nmol mg^–1^ protein min^–1^, cytochrome *b*_5_ was 379 nmol mg^–1^ protein, and P450 2C9 activity toward diclofenac 4′-hydroxylation was 2060 pmol mg^–1^ protein min^–1^. Several other P450 isoforms present in the HLM include P450 1A2, 2A6, 2C8, 2C19, 2D6, 2E1, and 3A4. The P450 contents were estimated by the standard carbon monoxide spectral assay, and the activities were characterized by the NADPH assay with standard P450 drug candidates, as detailed before.^19^ P450 2C9 expressed bactosomes were also purchased from XenoTech (Lenexa, KS, USA). The P450 2C9 concentration in the bactosomes was 190 pmol mg^–1^ protein and a total protein concentration of 12.6 mg mL^–1^, the bactosomal P450 2C9 activity toward diclofenac 4′-hydroxylation (*V*_max_) was 98 pmol min^–1^ pmol^–1^ 2C9 and the cytochrome *c* reductase activity was 1405 nmol min^–1^ mg^–1^ protein.

Bactosomes are bacterial membranes expressed with a specific recombinant P450 isoform. The bactosome expression system offers high levels of an individual P450 isoform, which is useful for characterizing the specific isoform of a new drug candidate or a biosensor that operates on a particular P450 isoform. Diclofenac used as a model P450 drug substrate, predominantly metabolized by the P450 2C9 isoform, was purchased from Sigma-Aldrich (St. Louis, MO, USA), and the 4′-hydroxydiclofenac metabolite standard was purchased from Cayman Chemicals (Ann Arbor, Michigan, USA). Amine-functionalized multiwalled carbon nanotubes (denoted as MWNT-NH_2_, purity: 95%, OD: 7–13 nm, length: 55 μm, amino (NH_2_): 0.45%) were purchased from US Research Nanomaterials, Inc. TX, USA. 1-Pyrenebutylamine (Py-NH_2_) was obtained from Toronto Research Chemicals, Ontario, Canada. All aqueous reagents were prepared in deionized water using a Milli-water purification system (Millipore Ltd., Billerica, Massachusetts, USA). All other reagents and chemicals used were of high-purity analytical grade.

### 2.2 Amine-Enriched Pyrenyl-Nanocarbon Electrode Modification

Due to phospholipids, HLM and P450 2C9 bactosomes are negatively charged.^19,26,40^ Therefore, to facilitate electrostatic interactions at physiological pH with positively charged amine end groups and large hydrophobic interactions with the high surface area nanotubes and pyrenyl moieties of the linkers, we designed the pyrenyl-nanocarbon electrode modification. In this, we dry-coated overnight the amine-functionalized MWNTs (10 μL, 1 mg mL^–1^ dispersion in dimethylformamide (DMF), obtained by ultrasonication at room temperature for 4 h) on the base graphite disk electrodes or carbon or gold electrode arrays, followed by strong pi–pi stacking for 1 h at room temperature with the pyrene groups of 10 mM 1-pyrene butylamine (20 μL) in DMF inside a closed moisturized beaker placed in a styrofoam box (to prevent the pyrenylamine solution from drying during the pi–pi stacking from the solution with the basal plane sidewalls of nanotube surface on the electrodes,^34,35^ a heterogeneous process). Following the modification, the electrodes were washed in deionized water and dried in nitrogen before adsorbing the as-received liver microsomes or 2C9 bactosomes.

### 2.3 Voltammetry

High-purity graphite disk (HPG, geometric area of 0.2 cm^2^, EDM Inc., MN, USA) electrodes were used for both cyclic voltammetry (CV) and rotating disk electrochemistry (RDE). For RDE experiments, an electrode rotation rate of 300 rpm (Eco Chemie Autolab rotator with a motor controller, Metrohm Inc., USA) was employed to determine the electrocatalytic oxygen reduction currents. Before each use, HPG electrodes were freshly polished on a P320 SiC-grit paper, sonicated for 10 s in ethanol and 10 s in water, and dried under nitrogen. For eight-electrode array-based electrochemical measurements of electrocatalytic oxygen reduction by the microsomal bioelectrodes, screen-printed carbon, and gold eight-electrode arrays (geometric area of each working electrode is 0.13 cm^2^), purchased from DropSens Inc., Spain, were used as single-use disposable arrays.

Electrochemical measurements were performed in a three-electrode glass electrochemical cell connected to an electrochemical workstation (CH Instruments, Model CHI 6017, Texas, USA). The three-electrode cell consisted of an Ag/AgCl reference electrode (1 M KCl, CH Instruments). A Pt-wire counter-electrode and polished HPG-rotating disk working electrodes were used as such or after pyrenylamine-amino nanocarbon modification. Similarly, eight-electrode gold or carbon screen-printed arrays (multi 8-channel potentiostat purchased from CH Instruments) were used for the pyrenylamine-amino nanocarbon modification, followed by the microsomal or bactosomal adsorption from solution for electrochemical studies.

### 2.4 Human Liver Microsomal and P450 2C9 Bactosomal Biofilm Electrodes

A biofilm of HLM on electrodes was prepared by placing 20-μL solution (used as received from the supplier) for 20 min on freshly polished HPG electrodes (denoted as HPG/HLM) or that modified with the pyrenylamine-amino nanocarbon chemistry (denoted as HPG/MWNT-NH_2_/Py-NH_2_/HLM), or a similar modification on eight-electrode gold and carbon arrays under ice-cold conditions to allow electrostatic and other secondary interactions of the membrane-bound P450 enzymes with the electrode surface. Similarly, P450 2C9 bactosomal films were also prepared for comparison. The unbound microsomal and bactosomal solutions were washed away from the electrode surface using deionized water to establish the strongly adsorbed biofilm layer for electrochemical measurements. CV was performed under an argon atmosphere to characterize the redox peaks of the microsomal bioelectrodes.^8^

### 2.5 Electrocatalytic Diclofenac Hydroxylation, Identification, and Quantification

Voltage-driven conversion of diclofenac to 4′-hydroxydiclofenac electrocatalyzed by the designed microsomal and 2C9 bactosomal bioelectrodes was confirmed by liquid chromatography–mass spectrometry (LC–MS). The bioelectrodes were immersed (4 electrodes of 0.2 cm^2^ geometric area each) in a beaker containing 100 μM diclofenac in 2 mL of 50 mM phosphate buffer, pH 7.0 containing 150 mM NaCl. Electrolysis was carried out for 1 h at an applied potential of −0.6 V vs Ag/AgCl in saturated oxygen phosphate buffer, pH 7.0. The reaction mixture was analyzed by LC–MS (LC column: premier C_18_ 3 μ 100 × 4.6 mm). High-performance liquid chromatography (HPLC) was used to identify diclofenac and its metabolite 4′-hydroxydiclofenac. A premier C18 column of 10 cm length purchased from Shimadzu Scientific Instruments, Inc. (Columbia, MD) was used with the Dionex UltiMate 3000 HPLC system (ThermoFisher Scientific, Waltham, MA). A mobile phase composition of 20% acetonitrile/80% water was used at a flow rate of 0.3 mL min^–1^. The diclofenac and 4′-hydroxydiclofenac peaks were identified based on the chromatograms of standards and mixtures run under identical conditions on the HPLC system.

## 3 Results and Discussion

**Figure 1:**
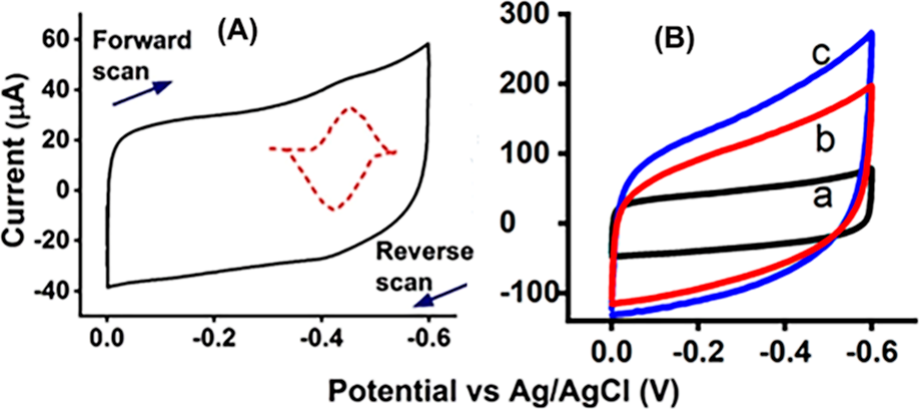
(A) Cyclic voltammograms of HPG/MWNT-NH_2_/Py-NH_2_/HLM biofilm (solid line, as acquired) and that of background-subtracted (broken line) eliminating the nonfaradaic charging currents to enable better visualization of the redox peaks. (B) (a) Cyclic voltammograms of polished HPG surface, (b) after dry coating MWNT-NH_2_ on a polished HPG electrode, and (c) after pi–pi stacking the HPT/MWNT-NH_2_ electrode with Py-NH_2_ showing no peaks and only charging or nonfaradaic currents in the absence of an immobilized HLM film under the same potential range. Experimental conditions: argon-purged phosphate buffer, pH 7.0, 25 °C, scan rate 0.3 V s^–1^.

Figure 1A represents the cyclic voltammograms of as acquired, and the background subtracted human liver microsomal biofilm adsorbed onto the amine-enriched pyrenyl-nanocarbon-modified HPG electrodes. The formal potential observed around −0.45 V vs Ag/AgCl (pH 7.0, 0.3 V s^–1^ scan rate) was consistent with prior reports on liver microsomal and P450 bactosomal biofilms on carbon and gold-self-assembled monolayer electrodes, which was identified to originate from the membrane-bound microsomal P450-reductase as the electron receiver from the electrode.^8,18,19^

Figure 1B(a–c) cyclic voltammograms infer that in the absence of an immobilized enzyme film, the voltammograms exhibit only the nonfaradaic capacitive currents for the polished HPG (a), after coating MWNT-NH_2_ (b), and after further pi–pi stacking with Py-NH_2_.

**Figure 2:**
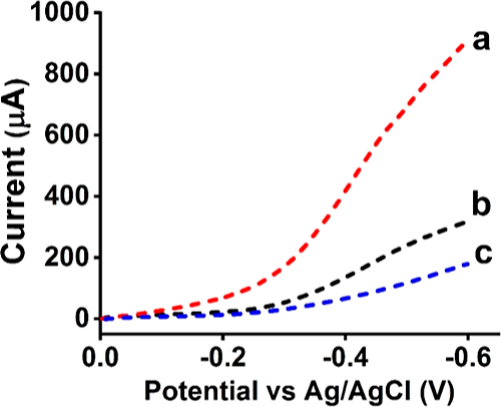
Rotating disc catalytic oxygen reduction voltammograms of (a) HPG/MWNT-NH_2_/Py-NH_2_/HLM, (b) HPG/HLM, and (c) polished HPG surface in saturated oxygen, phosphate buffer, pH 7.0, 25 °C, 300 rpm electrode rotation rate, and scan rate 0.3 V s^–1^.

Figure 2a–c presents the rotating disc oxygen reduction voltammograms electrocatalyzed by the designed HPG/MWNT-NH_2_/Py-NH_2_/HLM, HPG/HLM without the pyrenyl-nanocarbon modification, and only the polished bare HPG surface in saturated oxygen at 300 rpm electrode rotation rate at a scan rate of 0.3 V s^–1^ in phosphate buffer, pH 7.0, 25 °C. The polished bare HPG electrodes alone with no adsorbed HLM did not show a significant catalytic onset but with a flow of nonfaradaic charging currents at a more negative potential. The electrocatalytic oxygen reduction currents from the immobilized HLM films on the modified HPG/MWNT-NH_2_/Py-NH_2_ electrodes are ∼3-fold greater than those of the HLM film adsorbed on polished bare HPG electrodes. Moreover, the modified HPG/MWNT-NH_2_/Py-NH_2_/HLM bioelectrodes displayed a better electrocatalytic property from the lower overpotential onset of oxygen reduction compared to that of HPG/HLM electrodes. Observed electrocatalytic oxygen typical of heme enzymes such as P450s in liver microsomes suggests electron mediation through the P450-reductase molecules from the electrode (Figure 1 anaerobic noncatalytic CV data and prior literature) and then to the P450 heme centers to reduce the heme-Fe(III) to heme-Fe(II) to enable oxygen binding and its subsequent electrocatalytic reduction to peroxide yielding currents. We have proved this mechanistic pathway in our prior liver microsomal,^8^ and P450 bactosomal electrochemical studies,^19^ and the presently modified amine-enriched nanotube-pyrenyl bioelectrode surface undergoes a similar electrode to P450-reductase to P450 electron transfer pathway.

**Figure 3:**
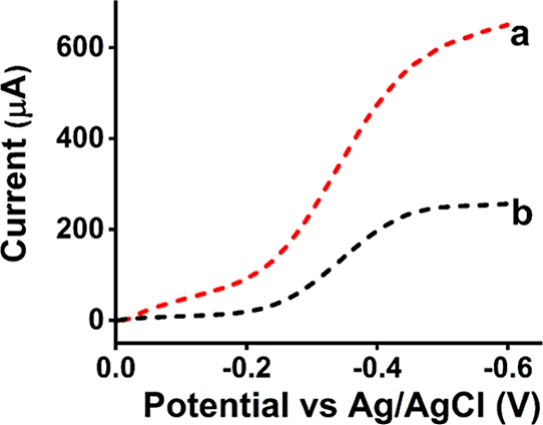
Rotating disc electrocatalytic oxygen reduction voltammograms of (a) HPG/MWNT-NH_2_/Py-NH_2_/P450 2C9 bactosomes and (b) HPG/P450 2C9 bactosomes in saturated oxygen, phosphate buffer, pH 7.0, 25 °C, 300 rpm electrode rotation rate, scan rate of 0.3 V s^–1^.

Following the liver microsomal electrocatalytic study, we designed polished HPG and HPG/MWNT-NH_2_/Py-NH_2_ modified electrodes adsorbed with a P450 2C9 bactosomal film. HLM contains a pool of various P450 isoforms, whereas the 2C9 bactosomes are recombinantly expressed with the specific isoform along with some coexpression of reductases (Experimental Section for details). Figure 3a,b shows the oxygen reduction electrocatalysis catalyzed by the 2C9 bactosomal film either absorbed on a polished HPG or modified with MWNT-NH_2_/Py-NH_2_. Similar to the HLM biofilm electrocatalysis, the 2C9 bactosomal films exhibited about a threefold catalytic current increase for the pyrenylamine-nanocarbon modified electrodes compared to the unmodified bulk HPG electrodes. More interestingly, by looking at the electrocatalytic voltammograms of HLM and 2C9 bactosomal films (Figure 2 vs Figure 3), we find notable differences in their shapes.

The HLM films showed a relatively linear response in the current–potential plot (Figure 2) compared to the prominent S-shaped electrocatalytic oxygen reduction voltammogram of C9 bactosomal film (Figure 3), suggesting a likely greater interfacial dispersion of enzyme redox centers in the HLM films and the associated electron transfer rates.^41^ Next, we examined the electrocatalytic conversion of diclofenac as a P450 drug candidate into 4′-hydroxydiclofenac by the designed liver microsomal and 2C9 bactosomal bioelectrodes on the HPG electrodes or that of eight-electrode arrays to test the approach with an array system as well as generate sufficient metabolites for identification and quantification. In this study, we used eight-electrode arrays of screen-printed carbon from DropSens Inc., modified with the amine-functionalized MWNT (MWNT-NH_2_) followed by 1-pyrenylbutylamine (Py-NH_2_) pi–pi stacking followed by the adsorption of a layer of HLM or 2C9 bactosomes solutions.

**Figure 4:**
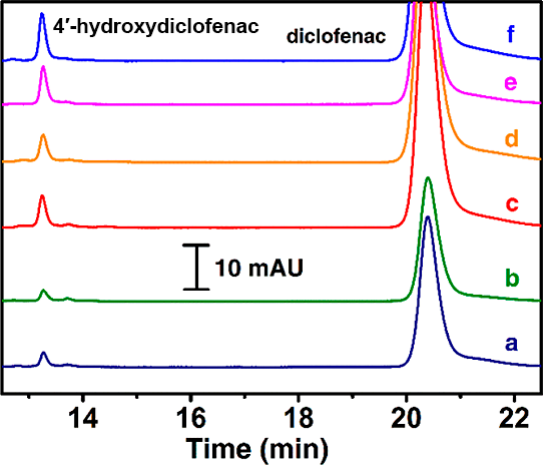
HPLC chromatograms of reaction mixtures (200 μM diclofenac) on (a) 4xHPG/HLM, (b) 4xHPG/2C9 bactosomes, (c) 8xCSPE/MWNT-NH_2_/Py-NH_2_/2C9 bactosomes, (d) 8xCSPE/MWNT-NH_2_/Py-NH_2_/HLM, (e) 8xAuSPE/MWNT-NH_2_/Py-NH_2_/2C9 bactosomes, and (f) 8xAuSPE/MWNT-NH_2_/Py- NH_2_/HLM.

Figure 4 corresponds to the electrocatalytic metabolite of diclofenac formed from the electrocatalysis by HLM or 2C9 bactosomes on eight-electrode arrays with the base surface of carbon (denoted as CSPE) or gold (denoted as AuSPE), and that of HPG disks used as four electrodes per the electrocatalytic drug conversion reaction to produce enough metabolites for identification. Using the standard 4′-hydroxydiclofenac chromatogram run under identical conditions, we determined its retention time as 13.2 min, and for the diclofenac drug, the retention time was 20.4 min (Figure S1, Supporting Information). Table 1 provides the estimated amounts of 4′-hydroxydiclofenac formed from the electrocatalysis of designed various HLM and 2C9 bactosomal biofilms assembled on modified HPG electrodes and screen-printed electrode arrays by using the calibration plot generated from the metabolite standards (Figure S1). The electrode modifications presented in Table 1 are used with various colored texts for quick distinction when viewed electronically.

**Table 1:**
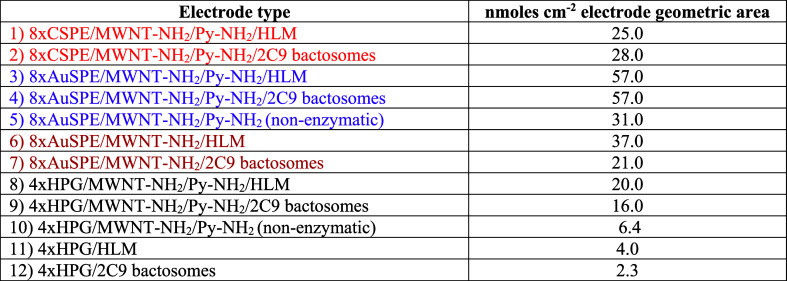
Electrode Modification and Amount of Electrocatalytic 4′-Hydroxydiclofenac Metabolite

electrode type	nmoles cm^–2^ electrode geometric area
(1) 8xCSPE/MWNT-NH2/Py-NH2/HLM	25.0
(2) 8xCSPE/MWNT-NH_2_/Py-NH_2_/2C9 bactosomes	28.0
(3) 8xAuSPE/MWNT-NH_2_/Py-NH_2_/HLM	57.0
(4) 8xAuSPE/MWNT-NH_2_/Py-NH_2_/2C9 bactosomes	57.0
(5) 8xAuSPE/MWNT-NH_2_/Py-NH_2_ (nonenzymatic)	31.0
(6) 8xAuSPE/MWNT-NH_2_/HLM	37.0
(7) 8xAuSPE/MWNT-NH_2_/2C9 bactosomes	21.0
(8) 4xHPG/MWNT-NH_2_/Py-NH_2_/HLM	20.0
(9) 4xHPG/MWNT-NH_2_/Py-NH_2_/2C9 bactosomes	16.0
(10) 4xHPG/MWNT-NH_2_/Py-NH_2_ (nonenzymatic)	6.4
(11) 4xHPG/HLM	4.0
(12) 4xHPG/2C9 bactosomes	2.3

The HLM and bactosomal 2C9 bioelectrodes displayed similar levels of 4′-hydroxydiclofenac formation on the pyrenylamine stacked amino-nanocarbon electrodes modified on carbon (Table 1, rows 1 and 2) or gold screen-printed electrodes (Table 1, rows 3 and 4). In the absence of immobilized HLM or 2C9 bactosomes, the modified gold screen-printed array electrode surface alone yielded almost 50% of metabolites due to the nonenzymatic electrocatalytic conversion likely arising from the metal impurities present in carbon nanotubes (Table 1, row 5). In the absence of 1-pyrene butylamine stacking on the MWNT-NH_2_ modification, the metabolite yields were significantly lower for the HLM and 2C9 bactosomal films (Table 1, rows 6 and 7), which suggests that the amine-functionalized pyrene linkers favor improved secondary interactions with liver microsomes or bactosomes, and this, in turn, favors an enhanced electroactive immobilization of the membrane-bound enzymes present in them. In the case of HPG disk electrodes modified with the pyrenylamine stacked amino-nanocarbon materials (Table 1, rows 8 and 9), the electrocatalytic metabolite yields were comparable between the HLM and 2C9 bactosomal films and are significantly greater than the nonenzymatic metabolite yield in the absence of any adsorbed biofilms (Table 1, row 10).

Without the pyrenyl-nanocarbon modification, HLM adsorbed onto polished HPG electrodes exhibited a 5-fold lower metabolite yield (Table 1, row 11, Figure S2 for comparative chromatograms), and that of 2C9 bactosomal film on polished HPG electrodes yielded ∼seven times lower metabolite (Table 1, row 12, Figure S3 for comparative chromatograms). Overall, the nonenzymatic production of the metabolite is less than the enzymatic microsomal and bactosomal-based electrocatalytic reactions. And the pyrenyl-nanocarbon-modified electrodes offer enhanced metabolite yields compared to other electrodes.

Table 2 provides a collective account of the presently designed pyrenylamine stacked amino nanotube-modified liver microsomal and 2C9 bactosomal electrodes with prior electrochemical reports on various other electrode designs and modifications. This representative collection of prior reports confirmed the observed reductase-specific formal potential and the P450-catalyzed drug hydroxylation in the designed microsomal and bactosomal bioelectrodes. In addition, Table 2 infers the range of various electrode materials, surface modifications, and P450 substrates utilized in making microsomal and bactosomal P450 bioelectrodes.

**Table 2:** Comparison of Various Representative P450 Liver Microsomal and Bactosomal Electrode Designs

electrode design	*E*°′ vs Ag/AgCl (mV)	oxygen reduction onset potential and current density (per geometric area) at –0.5 V subtracted for the nonenzymatic response	electrocatalytic drug conversion	refs
(1) (a) Au/cysteamine self-assembled monolayer (SAM) adsorbed with P450 2C9+reductase bactosomes	–450 ± 40 (pH 7.0)	–220 mV, 0.6 mA cm^–2^	diclofenac to 4′-hydroxydiclofenac	(19)
(b) Au/cysteamine SAM adsorbed with P450 2C9 bactosomes containing *no reductase* (please note the positive formal potential shift from (a) toward the P450 heme center and the lower O_2_ reduction current density)	–310 ± 20 (pH 7.0)	–120 mV, 0.16 mA cm^–2^		
(c) Au/cysteamine SAM adsorbed with only NADPH-reductase bactosomes containing *no P450 enzyme* (please note the reductase-centered more negative formal potential and negligible enzymatic O_2_ reduction currents in the absence of P450 enzymes)	–450 ± 38 (pH 7.0)	–224 mV, no net O_2_ reduction above the Au/cysteamine nonenzymatic background current since P450 is absent		
(2) HPG/MWNT-NH_2_/Py-NH_2_/HLM	–450 ± 20 (pH 7.0)	–260 mV, 3.0 mA cm^–2^	diclofenac to 4′-hydroxydiclofenac	this work
HPG/HLM	–450 ± 10 (pH 7.0)	–280 mV, 1.0 mA cm^–2^		
HPG/MWNT-NH_2_/Py-NH_2_/P450 2C9 bactosomes		–200 mV, 2.5 mA cm^–2^		
HPG/P450 2C9 bactosomes		–240 mV, 0.8 mA cm^–2^		
(3) PG/MWNT/HLM (MWNT here was not functionalized)	–460 ± 20 (pH 7.0)	–250 mV, 1.5 mA cm^–2^	testosterone to 6β-hydroxytestosterone	(27)
(4) HPG/HLM	450 ± 5 (pH 7.0)	–200 mV, 0.6 mA cm^–2^	testosterone to 6β-hydroxytestosterone	(8)
(5) amine-functionalized magnetic nanoparticles electrostatically assembled with P450 2C9+reductase bactosomes and adsorbed on graphite electrodes	–460 ± 10 (pH 7.0)	–270 mV, 0.9 mA cm^–2^	diclofenac to 4′-hydroxydiclofenac	(26)
(6) layer-by-layer polyion/HLM assembly on PG electrodes	–460 (pH 7.4)		4-(methylnitrosamino)-1-(3-pyridyl)-1-butanone bioactivation and DNA adduct detection	(42)
(7) P450 2C9 microsomes cast onto 4-aminothiophenol-modified Au electrode	–399 (pH 7.3)		tolbutamide to 4-hydroxytolbutamide	(43)
(8) self-assembled Au@MXene liver microsome electrochemical biosensor	–466 (pH 7.4)		rapid screening of aflatoxin B1 (0.01 to 50 μM linear range, detection limit 2.8 nM)	(44)

**Figure 5:**
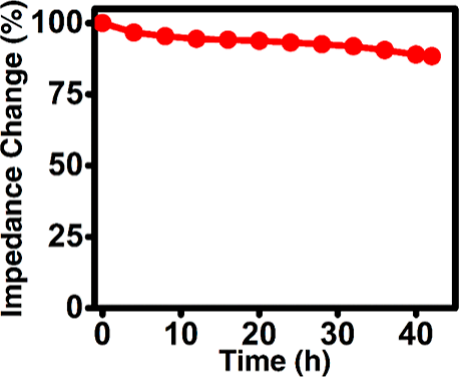
Biofilm stability assessment of the designed HPG/MWNT-NH_2_/Py-NH_2_/HLM bioelectrode by nonfaradaic impedance spectroscopy monitoring the % impedance change at an applied frequency of 5 Hz in pH 7.0 phosphate buffer at room temperature (23 °C).

The liver microsomal biofilm stability adsorbed on the HPG-modified pyrenylamine-stacked-amino carbon nanotube electrodes was monitored over 40 h by the nonfaradaic impedance spectroscopy monitoring the impedance change with time (in hours) at an applied low frequency of 5 Hz in pH 7.0 phosphate buffer at room temperature (23 °C) as shown in Figure 5. At such low-frequency regions, the electrode system exhibits a dominant double-layer capacitive behavior.^28,45^ Any biofilm loss is expected to change the interfacial double-layer charges and the associated capacitance change. Results indicate that the liver microsomal bioelectrode exhibits good stability as the relative impedance decrease over 40 h was less than 15% from the initial value. This stability is more than sufficient to utilize these bioelectrodes for electrocatalytic drug metabolism profiling and can also allow testing of their reusability for the same or different drugs and inhibitors, which will be examined in our future study. The corresponding impedance response curves over time are presented in Figure S4 (Supporting Information).

## 4 Conclusions

We demonstrated here the electrocatalytic drug conversion to metabolites by amine-functionalized pyrenyl and nanocarbon surface-modified electrodes immobilized with human liver microsomes or the recombinant single P450 2C9 expressed bactosomes. The amine chemical functionality enriched pyrenyl-carbon nanotubes offer enhanced electrocatalytic oxygen reduction currents and metabolite yield along with good surface stability of the electrostatically immobilized microsomes. The presence of various P450 isoforms in the human liver microsomes and their collective extent of biocatalytic activities likely facilitated or comparable metabolite yield than the single P450 2C9 bactosomes system. Size and orientational differences between the microsomal and bactosomal membranes on electrodes are other aspects to investigate on. Furthermore, detailed investigation from the materials science perspective of the designed bioelectrodes for reusability and scalability and a fundamental understanding of correlating the P450 and reductase expression levels with regard to their electrocatalytic performance forms our future study. In conclusion, the designed bioelectrodes have the potential to aid in the physicochemical analysis of novel drugs for metabolite profiling and P450 isoform identification and as biosensors for pollutant, drug, and chemical toxicity screening.
