# Supplementary material for: Nanobioelectrocatalysis Using Human Liver Microsomes and Cytochrome P450 Bactosomes: Pyrenyl-Nanocarbon Electrodes
Source: ACS Appl Bio Mater. 2024 Mar 3;7(4):2197–204. doi: 10.1021/acsabm.3c01170 (PMC11022171; doi:10.1021/acsabm.3c01170)
Supplement: Supplementary file 1 — mt3c01170_si_001.pdf [file mt3c01170_si_001.pdf]

Supporting Information

Nanobioelectrocatalysis using Human Liver Microsomes and Cytochrome P450 Bactosomes: Pyrenyl-Nanocarbon Electrodes

Gayan Premaratne, Jinesh Niroula, James T Moulton, and Sadagopan Krishnan*

Department of Chemistry, Oklahoma State University, Stillwater, Oklahoma 74078, United States

Corresponding Author: *E-mail: gopan.krishnan@okstate.edu

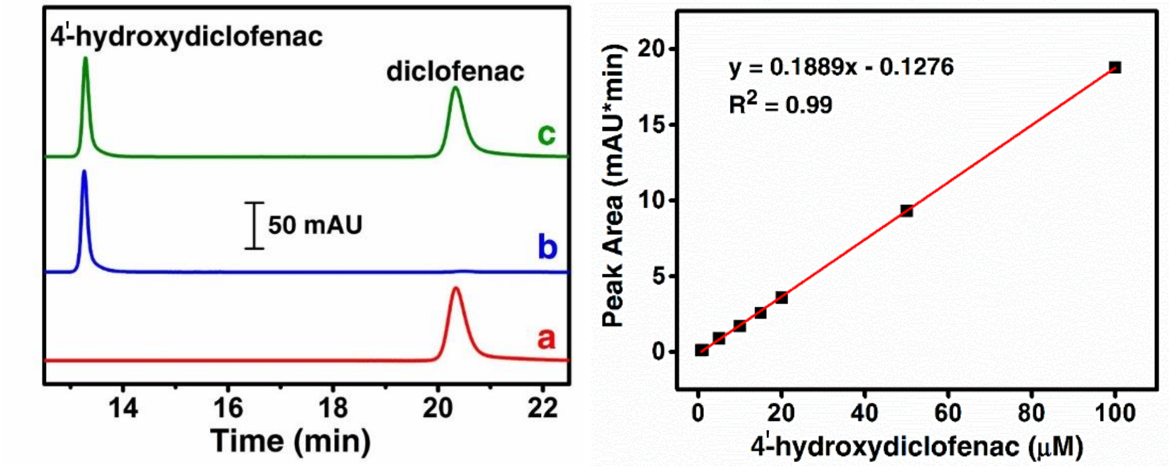

Figure S1. HPLC chromatograms of standards: On the left: **(a)** 200 µM diclofenac prodrug, **(b)** 100 µM 4'-hydroxydiclofenac metabolite, and **(c)** a mixture of 200 µM diclofenac and 100 µM 4'-hydroxydiclofenac. The retention time for standard 4-hydroxydiclofenac is 13.2 minutes, and for diclofenac, it is 20.4 minutes. The calibration plot with standard 4'-hydroxydiclofenac concentrations is shown on the right.

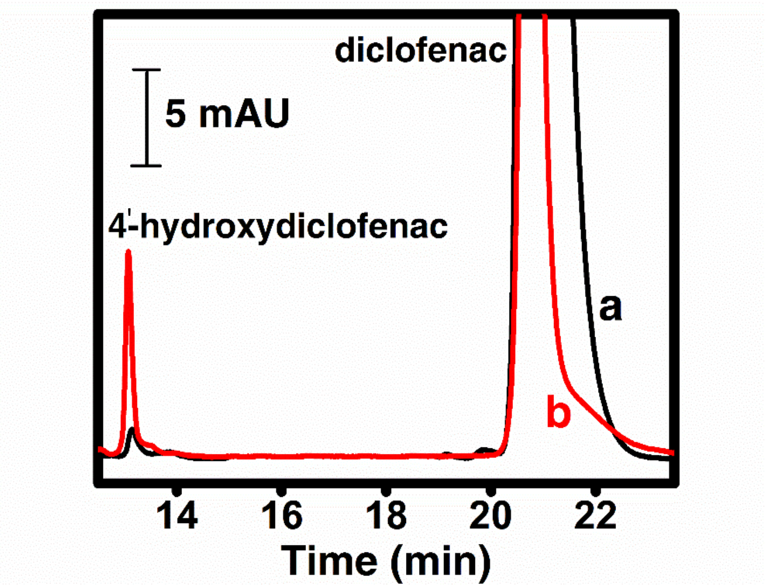

Figure S2. HPLC chromatograms of **(a)** 4xHPG/HLM bioelectrodes and **(b)** 4xHPG/MWNT-NH_2/Py-NH_2/HLM bioelectrodes. The reaction was performed with 200 µM diclofenac in 50 mM phosphate buffer containing 150 mM NaCl, pH 7.0, 23 ˚C with an applied potential of -0.6 V for 1 h. The chromatogram shows the peaks for the reactant, 200 µM diclofenac, at a retention time of 20.4 minutes and its major metabolic product, 4'-hydroxydiclofenac, at a retention time of 13.2 minutes. The estimated metabolite concentrations were 4.0 and 20.0 nmoles cm^{-2} electrode geometric area for **(a)** and **(b),** respectively, as presented in Table 1 in the manuscript.

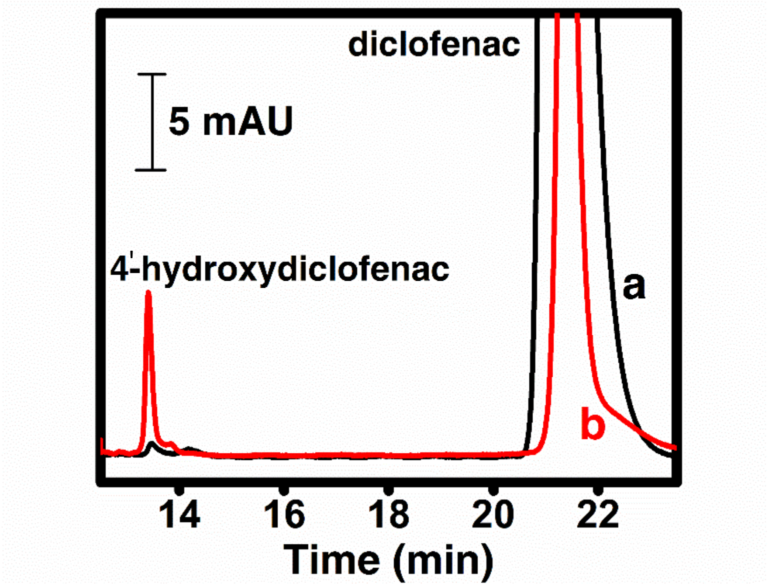

Figure S3. HPLC chromatograms of metabolite reaction mixtures electrocatalyzed by **(a)** 4xHPG/2C9 bactosomes and **(b)** 4xHPG/MWNT-NH_2/Py-NH_2/2C9 bactosomes electrodes. The reaction was performed with 200 μM diclofenac in 50 mM phosphate buffer containing 150 mM NaCl, pH 7.0, 23 ˚C with an applied potential of -0.6 V for 1 h. The estimated metabolite concentrations were 2.3 and 16.0 nmoles cm^{-2} electrode geometric area for **(a)** and **(b),** respectively, as presented in Table 1 in the manuscript.

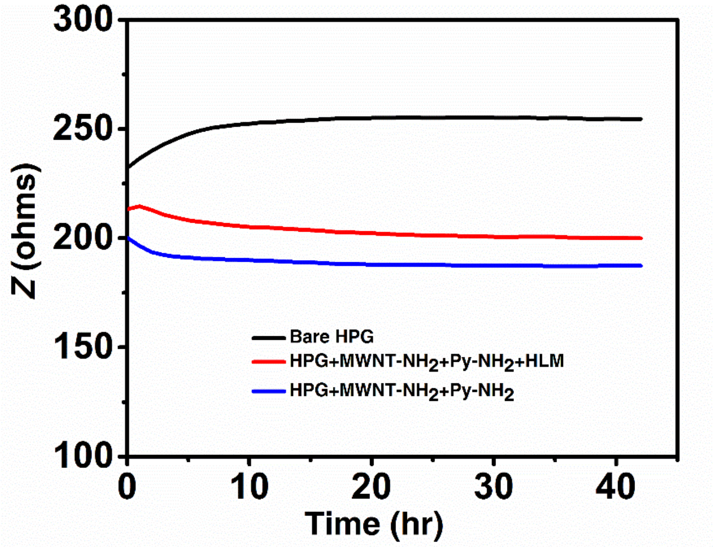

Figure S4. Non-faradaic impedance change was monitored for polished bare HPG, HPG/MWNT-NH_2/Py-NH_2/HLM modified electrode, and HPG/MWNT-NH_2/Py-NH_2 electrode without the HLM adsorption at 5 Hz with the time of exposure in pH 7.0 phosphate buffer at room temperature (23 ^{o}C).
